# Poly(3-hydroxybutyrate) 3D-Scaffold–Conduit for Guided Tissue Sprouting

**DOI:** 10.3390/ijms24086965

**Published:** 2023-04-09

**Authors:** Irina I. Zharkova, Aleksey V. Volkov, Aleksandr A. Muraev, Tatiana K. Makhina, Vera V. Voinova, Valentina M. Ryabova, Yulia V. Gazhva, Alena S. Kashirina, Aleksandra V. Kashina, Garina A. Bonartseva, Vsevolod A. Zhuikov, Konstantin V. Shaitan, Mikhail P. Kirpichnikov, Sergey Yu. Ivanov, Anton P. Bonartsev

**Affiliations:** 1 Faculty of Biology, M.V. Lomonosov Moscow State University, Leninskie Gory, 1-12, Moscow 119234, Russia; iblkr@mail.ru (I.I.Z.);; 2 Federal State Budgetary Institution “N.N. Priorov National Medical Research Center of Traumatology and Orthopedics”, Ministry of Health of the Russian Federation, Priorova Str. 10, Moscow 127299, Russia; alex.volkoff@gmail.com; 3 Department of Oral and Maxillofacial Surgery and Surgical Dentistry, Medical Institute, RUDN Universiry, Miklukho-Maklaya Str., Moscow 6117198, Russia; muraev@gmail.com (A.A.M.);; 4 A.N. Bach Institute of Biochemistry, Research Center of Biotechnology of the Russian Academy of Sciences, Leninsky Ave. 33, Bld. 2, Moscow 119071, Russia; 5 Federal State Budgetary Educational Institution of Higher Education “Privolzhsky Research Medical University”, Ministry of Health of the Russian Federation, Minin and Pozharsky pl., 10/1, Nizhny Novgorod 603005, Russia; 6 Department of Oral and Maxillofacial Surgery, Sechenov University, Trubetskaya Str., 8-2, Moscow 119991, Russia

**Keywords:** scaffolds, poly(3-hydroxybutyrate), conduit, mesenchymal stem cells, 3T3 fibroblasts, biocompatibility, tissue sprouting, older rats

## Abstract

Scaffold biocompatibility remains an urgent problem in tissue engineering. An especially interesting problem is guided cell intergrowth and tissue sprouting using a porous scaffold with a special design. Two types of structures were obtained from poly(3-hydroxybutyrate) (PHB) using a salt leaching technique. In flat scaffolds (scaffold-1), one side was more porous (pore size 100–300 μm), while the other side was smoother (pore size 10–50 μm). Such scaffolds are suitable for the in vitro cultivation of rat mesenchymal stem cells and 3T3 fibroblasts, and, upon subcutaneous implantation to older rats, they cause moderate inflammation and the formation of a fibrous capsule. Scaffold-2s are homogeneous volumetric hard sponges (pore size 30–300 μm) with more structured pores. They were suitable for the in vitro culturing of 3T3 fibroblasts. Scaffold-2s were used to manufacture a conduit from the PHB/PHBV tube with scaffold-2 as a filler. The subcutaneous implantation of such conduits to older rats resulted in gradual soft connective tissue sprouting through the filler material of the scaffold-2 without any visible inflammatory processes. Thus, scaffold-2 can be used as a guide for connective tissue sprouting. The obtained data are advanced studies for reconstructive surgery and tissue engineering application for the elderly patients.

## 1. Introduction

Biodegradable polymers are gaining more and more extensive applications: food packaging [1], beauty products, agriculture, medical devices, and consumer electronics [2]. Biodegradable polyesters (polylactic acid (PLA), poly(lactic-co-glycolic acid) (PLGA), poly(ϵ-caprolactone) (PCL), poly(3-hydroxybutyrate) (PHB), poly(3-hydroxybutyrate-co-3-hydroxyvalerate) (PHBV), etc.) are becoming increasingly noticeable on the market of materials for regenerative medicine [3] due to their wide range of appropriate and suitable biological and physicochemical properties as materials for medical implant manufacturing [4]. However, there are studies revealing the toxicity of some materials used in medicine (based on PLA) after prolonged implantation [5], as well as the immunogenicity of some synthetic polyesters [6].

A number of scientific papers on the study of biodegradable polyester PHB, a most representative member of the natural poly(3-hydroxyalkanoates) (PHAs) family, increases from year to year. PHB and its copolymers are produced by microorganisms, which causes it to be possible to obtain a product with a wide variability of physicochemical properties by adjusting the cultivation parameters, cultural medium composition, and other conditions [7,8]. Regarding the industrial production of PHB, the global PHA market report published in August 2022 by Technavio (a renowned global technology research and consulting company) announced an increase in the commercial turnover of PHAs due demand for biodegradable plastics and the sustainable production of PHAs [9]. By virtue of a good combination of mechanical and thermoplastic properties, PHB and its copolymers are applied to produce a line of products for various medical purposes: microspheres [10,11], 3D scaffolds [12,13], electrospun membranes, wound coverings, etc. [14]. PHB is also often used as a basic compound of composite materials [15,16,17,18]. PHB has a number of unique biological properties: complete biodegradability to non-toxic products, biocompatibility, non-carcinogenicity, and special diffusion properties that provide sustained drug release [19,20,21,22]. PHB undergoes hydrolytic, enzymatic, and cellular biodegradation, and the degradation time is highly dependent on the molecular weight, crystallinity degree, and device shape and microstructure [23]. PHB is also used as biomaterial to manufacture tissue-engineering scaffolds for cell cultivation, including growing mesenchymal stem cells (MSCs) [20].

Scaffold-guided tissue engineering has two main approaches: (a) developing a scaffold as a barrier for tissue growth and (b) developing a scaffold as a conduit for tissue sprouting. With a scaffold barrier, the tissue must either properly integrate with the scaffold surface or simply prevent the tissue from sprouting in the undesirable direction. With a scaffold–conduit, on the other hand, the tissue should sprout in the desired direction. Of course, these scaffolds must have a completely different shape and microstructure. The non-toxicity of these scaffolds is, of course, important in both cases, but the presence of increased biocompatibility is more significant in the case of the scaffold–conduit [3,20,24].

There are a series approaches to manufacture scaffolds and other medical devices for tissue engineering: phase separation, emulsion freeze drying, fiber bonding, gas foaming, electrospinning, rapid prototyping (including 3D-printing), salt leaching, etc. For guided sprouting and the regeneration of a particular tissue, it is highly important to consider the microstructure and pore-inner-surface nanotopography of the scaffold. These morphological properties are extremely important factors in choosing the appropriate technique for use in each case. Therefore, to date, the problem of the relationship between scaffold micro- and nanostructures and cell/tissue integration with scaffolds remains relevant despite a huge number of investigations [24].

In this work, we subcutaneously implanted two types of PHB scaffolds with different shapes and microstructures and assessed the degree of penetration of the loose connective tissue in depth. Thus, the purpose of this study is to compare two PHB 3D scaffolds with different shapes and microstructures: the in vitro cell growth, as well as in vivo guided tissue sprouting.

## 2. Results and Discussion

### 2.1. Design and Microstructure of Scaffolds

**Figure 1:**
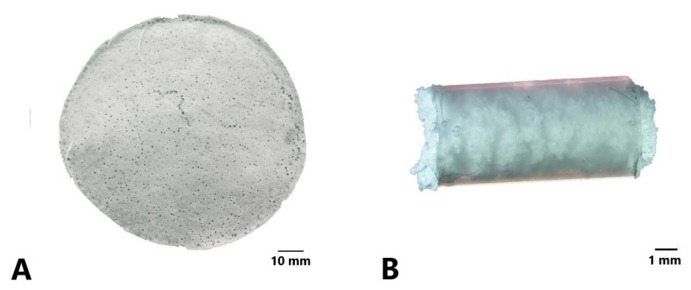
The photo images of porous PHB scaffolds. (**A**) Scaffold-1. (**B**) Scaffold-2.

The images of the obtained scaffold-1 and scaffold-2 are shown at Figure 1. Thus, the design of the devices differs dramatically: scaffold-1 is a flat device with two sides that have various microstructures; scaffold-2 is a two-part barrel-shape device that consists of a polymer tube filled with a 3D-scaffold.

**Figure 2:**
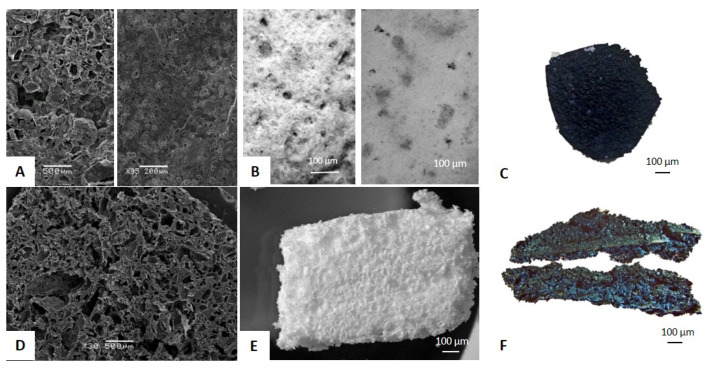
The images of porous PHB scaffolds: (**A**, **left**) the external side of scaffold-1, SEM, ×30; (**A**, **right**) the internal side of scaffold-1, SEM, ×35: (**B**, **left**) the external side of scaffold-1, WLM; (**B**, **right**) the internal side of scaffold-1, WLM; (**C**) scaffold-1, ink test, WLM; (**D**) scaffold-2, cross-section; SEM, ×30; and (**E**) scaffold-2, cross-section; WLM; (**F**) scaffold-2, ink test, WLM.

Therefore, scaffold-1 may act as a 2D-barrier, whereas scaffold-2 may serve as a 3D construct to guide the tissue germination. The study of the scaffold samples using scanning electron microscopy (SEM) and wide-field light microscopy (WLM) (Figure 2) showed that both types of scaffolds had irregular pores with unique inner surface structures. The internal microstructure of the PHB 3D scaffolds obtained using the one- and two-step leaching techniques differed greatly. These varieties are explained by the use of different porogens with different particle sizes.

To manufacture both types of scaffolds, we used ammonium carbonate, which decomposes when heated. This chemical reaction is fast and intense, so that pores are formed not only by salt leaching but also by the gas foaming. For scaffold-1, the pores emerged by breaking the polymer surface, so they are called “rupture pores”. At the same time, the porosity was also formed by folds of the polymer matrix (“lacunae”). Flat scaffolds have two sides: the side bordering with the Petri dish glass we call “internal” and the other “external”. Comparing the two sides of scaffold-1, it can be seen that the external side during manufacture has irregular pore morphology (Figure 2A,B left), while the side facing the glass substrate is smoother (Figure 2A,B right). Thus, this method allows for obtaining a flat, porous structure, one side of which is smooth and the other rough. Due to there being small pores on the smooth internal size of scaffold-1, it can be used in some cases as a barrier for the growth of certain cell types, e.g., fibroblasts [25].

The additional use of sucrose as a second leaching agent for scaffold-2 preparation led to the formation of 3D scaffolds of given shapes and complex microstructures with rupture pores and lacunae (Figure 2D,E). The obtained internal and outer porous structure of scaffold-2 was uniform without significant difference in pore size and shape between the “internal” and “external” sides. Moreover, the use of sucrose crystals with selected sizes allowed for regulating the pore size of scaffold-2. Scaffold-2 was also placed in a PHB/PHBV conduit to provide the guided tissue germination. Both scaffolds have an interconnected pore structure, which was shown using an ink test: closed pores in the scaffold in the form of black spots on the cross-section of scaffold-1 and scaffold-2 were absent (Figure 2C,F). Both obtained scaffolds are suitable for cell growth. Their average pore size is shown in Table 1. The pore size of the PHB scaffold-1s on the “internal” side ranged from about 10 to 50 μm, while, on the “external” side, it is from about 100 to 350 μm. The samples of scaffold-2 have pore sizes between approximately 30 and 400 μm (Table 1). The scaffold porosity was calculated according to [26] and shown in Table 1. The calculations showed that the average porosity of the PHB scaffold-1s was 87 ± 7%, while the average porosity of the scaffold-2s was 94 ± 3% (Table 1). The thermophysical properties of both scaffold types almost did not differ (Table S1, Supplemental Materials).

The scaffolds with mean pore sizes ranging from 20 μm to 1500 μm were used in the bone tissue engineering applications [27]. It was also shown that the bioactive material with approximately 100 μm pores was appropriate for cell migration and nutrient transport [28,29]. In the work of Peyton et al., the pore size from 7 to 17 µm was studied, which is preferred by MSCs, and it turned out that the highest probability for substantive cell movement through the pores was observed for the mean pore diameter of about 12 µm [30]. As for the soft tissues, for example, the gingival epithelial tissue optimally regenerated with a matrix pore size of about 100 μm [31], and the MSCs under conditions of adipogenic differentiation grew well and proliferated on the scaffolds with pore sizes ranging from 200 to 580 μm [32].

### 2.2. Cell Growth in Scaffolds

**Figure 3:**
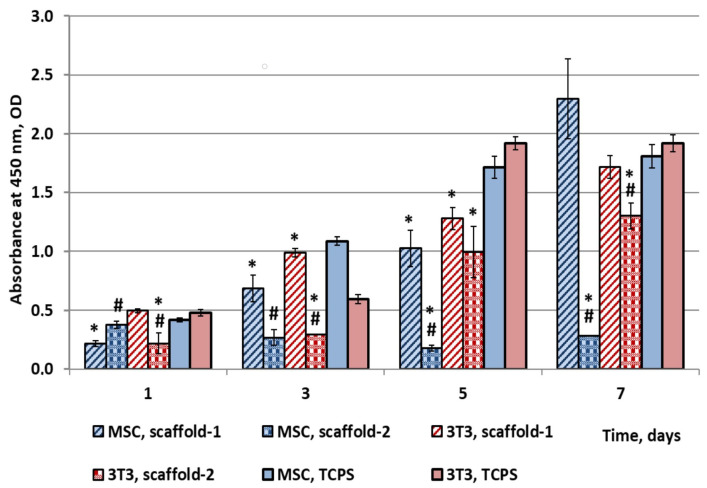
Adhesion and cell proliferation of MSCs and fibroblasts on scaffolds measured using XTT test; *n* = 6; * *p* < 0.05: scaffold-1 and scaffold-2 vs. TCPS; # *p* < 0.05: scaffold-2 vs. scaffold-1 for MSC and 3T3 fibroblasts, respectively.

The data on cell attachment and growth examination are shown in Figure 3. It was observed that the scaffold-1s are more suitable for cell growth and proliferation on the 3rd, 5th, and 7th days in both cases of MSCs and 3T3 fibroblasts compared to scaffold-2s (Figure 3). However, it is noticeable that the 3T3 cells grew on scaffold-2s much better than the MSCs, which was apparently due to the structure of the scaffold. Ashworth et al. [33] argue that fibroblasts prefer more structured pores, oriented toward the interior of the scaffold, so the shape and microstructure of scaffold-2 is probably more suitable for their growth and proliferation.

**Figure 4:**
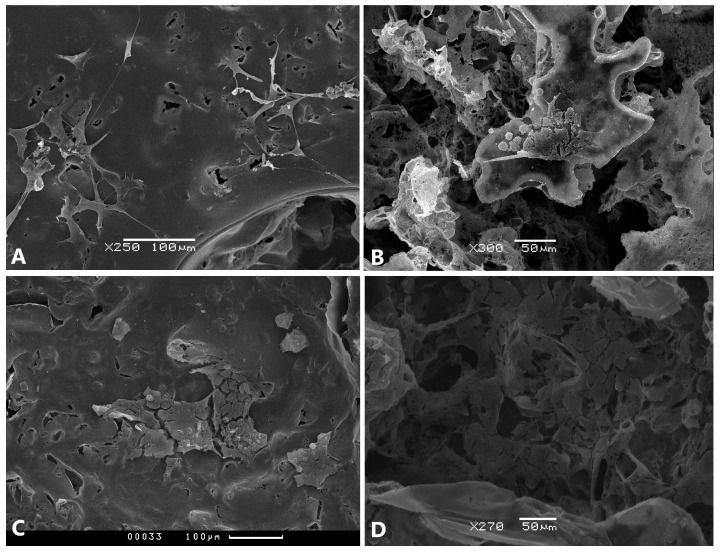
The SEM images of cell attachment and growth on scaffolds. (**A**) MSCs on scaffold-1s; (**B**) and MSCs on scaffold-2s; (**C**) 3T3 fibroblasts on scaffold-1s; (**D**) 3T3 fibroblasts on scaffold-2s. ×250–300. Original microphotographs (**A**–**D**) in good resolution are presented in Supplemental images for Figure 4. Histological evaluation of tissue reaction to PHB films as Control is presented in Supplemental Materials, Figure S3.

Our previous studies have shown that MSCs are capable of long-term cultivation on type 1 scaffolds and can withstand up to several passages without a change in phenotype [34]. In another previous study [13], the MSCs proliferated on the scaffolds that were similar to scaffold-2s, also being very slow, and they were associated with an initiation of their spontaneous osteogenic differentiation. We suggest that, in this case, too, the structure of the scaffold-2 pores could promote spontaneous osteogenic differentiation, which initiated the inhibition of cell proliferation. In the case of non-stem cells, fibroblasts, this effect did not manifest. Thus, it can be assumed that scaffold-1s are suitable for the growth and proliferation of MSCs, while the scaffold-2s are more suitable for other purposes: fibroblasts sprouting and osteogenic differentiation of MSCs. The data obtained using SEM visually demonstrate the MSCs (Figure 4A,B) and 3T3 fibroblasts (Figure 4C,D) attachment and spreading on polymer scaffolds on 6th day of cultivation. The SEM data were confirmed by a CLSM study of MSC attachment and growth on scaffold-2s (Supplemented Materials, Figure S2).

Moreover, cell growth is dependent not just on the shape and microstructure of scaffolds but also and mainly on the micro- and nanotopography of the inner pore surface. The choice of polymer template fabrication method must therefore be carefully considered, since, for example, 3D-printing and gas foaming methods produce completely different surface micro- and nanotopographies, while the scaffold shape and pore size can be the same [35]. In Schulte et al., NIH L929 fibroblasts demonstrated a notable unidirectional movement along the PEG–hydrogel channels with a diameter of 5–15 μm [36]; Lizarraga-valderrama et al. demonstrated a direct correlation between the diameter of the PHB microfibers of electrospun scaffolds (about 3 and 13 μm) and neuronal cells growth and differentiation [37]. In the work of Nam et al., there is a good confluence of rat hepatocytes on PLLA scaffolds with pores of 300–400 μm [38]; stem cells of different origins grew and proliferated differently on surfaces of different topographies with the linear size of teh topographical elements ranging from tens of nanometers to micrometers [39].

### 2.3. Tissue Reaction Study

**Figure 5:**
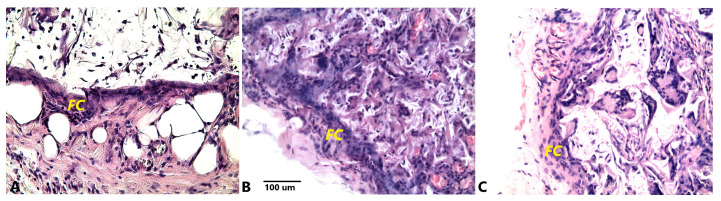
Histological evaluation of tissue reaction to scaffold-1 subcutaneous implantation on 7th (**A**), 14th (**B**), and 60th (**C**) days. The yellow mark FC shows the fibrous capsule formation. Hematoxylin-eosin staining; ×200. Original microphotographs (A, B, and C) in good resolution are presented in Supplemental images for Figure 5. Histological evaluation of tissue reaction to PHB films as Control is presented in Supplemental Materials, Figure S3.

The implantation of scaffold-1 was carried out subcutaneously on the right and left of the white line; the autopsy was performed 7, 14, and 60 days after the surgical procedure (Figure 5). No macroscopic signs of acute inflammation and septic foci were detected at these times at autopsy. In the histological sections, the capsule formation was observed: it had a thickness of 100–150 μm, which reached a final size of 300 μm at 60 days of implantation. It was also observed that the surrounding tissues germinated inside the implant.

**Figure 6:**
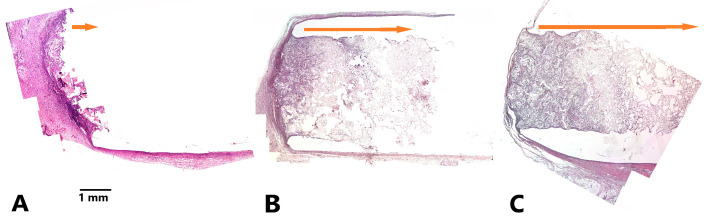
Histological evaluation of tissue reaction to scaffold-2 subcutaneous implantation on 7th (**A**), 14th (**B**), and 28th (**C**) days. Gradual germination of connective tissue into the cavity of the tubule along scaffold matrix can be observed. The arrow shows the direction of germination of loose connective tissue. Hematoxylin-eosin staining ×100. Original microphotographs (**A**–**C**) in good resolution are presented in Supplemental images for Figure 6.

During the histological research, on the 7th day of the tube-form scaffold-2 subcutaneous implantation, it was found out that biomaterial was surrounded by fibrous connective tissue with small infiltration by lymphocytes (Figure 6A). At both ends of the implanted tube-form scaffold-2, the initial signs of connective tissue ingrowth were observed. The young connective tissue mainly contained lymphocytes and a few macrophages.

During the histological research, on the 14th day of implantation, it was found that the smooth surface of scaffold-2 was surrounded by mature fibrous connective tissue. Both ends of the tubular scaffold showed signs of young connective tissue sprouting up to 1/6 of its volume, whereas both ends of the scaffold were already filled with mature connective tissue. On the 28th day of implantation, the germination of young connective tissue up to 1/3 of its volume were demonstrated from both ends of the tube-form scaffold-2. The scaffold was filled with loose connective tissue of different degrees of maturity: the young connective tissue was in the middle and the matured tissue was at the ends. The young connective tissue mainly contained lymphocytes and numerous macrophages (including foreign body-type giant cells). In turn, the matured loose connective tissue consisted of fibroblasts and lymphocytes, but neutrophils were not detected. As a result of our histological studies, it was revealed that, during the implantation of the PHB-based scaffolds, the implant failure or allergic reactions to the implanted material was not observed. The inflammatory process should be characterized as a typical reaction to a foreign body—granulomatous inflammation. With regard to the resorptive properties of the PHB biomaterial, the walls (outer contour) of the conduit had a minimal capacity for resorption, while the 3D-scaffold inside was more susceptible to it; the biodegradation of the scaffold-1 was minimal. The obtained data showed marked differences in the tissue response to the implanted scaffold, which obviously cannot be related to the PHB itself as a material, but to its microstructure and, above all, simply to its shape. If loose connective tissue is allowed to grow through the porous scaffold, the tissue response to such a scaffold will be much milder than to a scaffold–barrier with a structured surface. It can be suggested that a structured surface (e.g., porous or with high roughness) provokes fibrous tissue to sprout, whereas, if the shape of the scaffold is, in contrast, a barrier, a tissue response occurs with a fibrous capsule forming around it. If the shape of the scaffold promotes tissue germination, sprouting will occur and no insulating fibrous capsule will form. It should also be noted that, probably due to the use of older rats, we obtained different tissue response effects for the PHB scaffolds of different shapes and microstructures. In the young rats that are usually used research, the tissue response to the two types of scaffold would probably be equally mild, considering the high biocompatibility of PHB [21].

## 3. Materials and Methods

### 3.1. Polymer Production

PHB (molecular weight (Mw) = 150 kDa) was produced using Azotobacter chroococcum strain 7B, which was isolated and purified for biomedical application as previously reported [8]; the PHB Mw was determined using gel permeation chromatography (GPC) and viscosimetry [8].

### 3.2. Biopolymer Scaffolds Preparation

We produced 3D scaffolds using two different techniques: the first one was prepared using a one-stage salt leaching method with ammonium carbonate as the porogen (scaffold-1); the second one was manufactured using two-stage leaching technique with ammonium carbonate and sucrose as the porogens (scaffold-2). Scaffold-1 was prepared by modifying the salt leaching method, which is widely used for the manufacture of porous polymer scaffolds in tissue engineering [38]. This modification was based on the decomposition of solid salt under high temperatures, whereas the regular method requires the salt solution in an appropriate solvent. As a porogen, we used ammonium carbonate because of its possibility to decompose to (NH${}_{3}$, CO${}_{2}$, and H${}_{2}$O). The particles of ammonium carbonate of 94–315 μm size were mixed with 3% (*w*/*v*) PHB chloroform solution at a weight ratio of from 5 to 1, respectively. The salt dispersion in the polymer solution was poured on flat glass (Petri dish) with subsequent solvent evaporation. Then, the polymer scaffold was placed in hot (60 °C) water to create pores by ammonium carbonate decomposing, washed five times, and dried for 24 h. The scaffold-2s were manufactured using a novel modification of the salt leaching method: the two-stage salt leaching technique. A PHB solution in dichloromethane (EKOS-1, Moscow, Russia) with a concentration of 90 mg/mL was filled with two different porogens: using ammonium carbonate (Chimmed, Moscow, Russia) as the blowing agent and sucrose (Merck (Sigma-Aldrich), St. Louis, MO, USA) as the leaching agent in a ratio of 1:6 (*v*/*v*) each. The size of the crystals of the ammonium carbonate and sucrose were 40–94 μm and 94–315 μm, respectively. The sizes of the salt crystals were normalized using laboratory sieves, U1-ESL (Kraft, Chelyabinsk, Russia). The salt dispersion in the polymer solution was poured in a 5 mL glass vial with subsequent solvent evaporation. Then, the polymer scaffold was placed in hot (60 °C) water to create pores by performing ammonium carbonate decomposing and sucrose leaching, washing them five times, and drying for 24 h.

### 3.3. Polymer Conduits for Scaffolds Preparation and Scaffold–Conduit Plug-in

To obtain the biomaterial for polymer conduit production, a PHB/PHBV blend was preliminarily prepared. For this purpose, 3% (*w*/*v*) polymer solutions of PHB and PHBV in chloroform were mixed in a mass ratio 1:1. The polymer conduits were manufactured by performing multiple layer-by-layer casting from this PHB/PHBV blend solution using a rotating stainless steel drum with a 2 mm diameter with subsequent chloroform evaporation. Then, the drum with the deposited polymer was placed in water and a polymer cylinder was carefully removed from the drum. The obtained PHB/PHBV cylinders–conduits had walls that were 100 μm thick as measured using the electronic caliper (Krino, Monticello Brianza, Italy). Finally, the porous scaffold-2s obtained at the previous stage were cut and placed in these polymer conduits in the manner to fill the space inside the conduit as full as possible.

### 3.4. Microscopy

The macro-images of the scaffolds were taken using a Cyber-shot DSC-RX100 digital camera (Sony, Tokyo, Japan) with the Macro function. The microstructures of the scaffolds were studied by performing WLM and SEM using a stereomicroscope Nikon SMZ1500 (Nikon, Tokyo, Japan) and a scanning electron microscope JSM-6380LA (JEOL, Tokyo, Japan), respectively. The scaffolds with attached MSCs and 3T3 cells were also examined using SEM with the special pretreatment of specimens as previously reported [16].

The scaffold porosity was calculated according to [26]. The PHB bulk density was 1.243 g/cm^3^. The mass was measured on the scale AL-64 (Sartorius (Acculab), Göttingen, Germany). The scaffold thickness was measured using a caliper (Krino, Monticello Brianza, Italy). We used Equations (1) and (2) to calculate the apparent densities and porosities:

(1) $$Apparent\;density\;\left({g/\mathrm{cm}}^{3}\right)=\frac{Mass\;of\;scaffold,\;g}{Membrane\;thickness,\;\left(\mathrm{cm}\right)\;\times \;area\;\left({\mathrm{cm}}^{2}\right)}\;,$$

(2) $$Porosity\;\left(\%\right)=\frac{Apparent\;density\;({g/\mathrm{cm}}^{3})}{Bulk\;density\;of\;membranes\;({g/\mathrm{cm}}^{3})}\;.$$

Image J software was used for the SEM and WLM image processing. The data values are presented as an average (*n* = 15). To determine the interconnection of the pores, the scaffold-1 samples were immersed in ink solution and dried, while the scaffold-2 samples were immersed in ink solution (Sanford L.P., Atlanta, GA, USA), dried, and cross-sectioned. Then, the effectiveness of the ink impregnation of the scaffold pores and the presence of unimpregnated areas (closed pores) was examined using WLM [40].

### 3.5. In Vitro Cell Viability Test

The MSCs and 3T3 cells are among the most commonly used cultures for testing biomaterials and medical devices [41,42]. To study cell growth on scaffolds in vitro, the primary rat bone marrow MSC culture and mouse fibroblast cell line 3T3 were used. We used a biocompatibility in vitro test to estimate the cell attachment to the scaffolds and its growth on the scaffolds. The 3T3 cells (Biolot, Sankt-Peterburg, Russia) were maintained in DMEM, supplemented with 10% FBS and 50 U/mL penicillin/streptomycin. The bone marrow MSCs were isolated from the femurs of young (3–8 days old) Wistar rats as previously described [16,43]. The isolated MSCs were cultured for 2 weeks in α-MEM (PanEco, Moscow, Russia) supplemented with 10% fetal calf serum (FCS, Biological Industries, Beit Haemek, Israel), 50 U/mL penicillin/streptomycin. The cells were incubated at 37 °C in a 5% CO_2_ incubator and the medium was changed every 3 days. The phenotype of the isolated MSCs was verified with the cell surface markers CD90, CD45, CD29, and CD11 b/c (Thermo Fisher Scientific (eBioscience), Waltham, MA, USA) (Figure S1, Supplemental Materials).

For the cytotoxity testing, the first cells were detached using trypsin/EDTA (PanEco, Russia) and a 100 μL cell suspension, including 2000 cells, was plated in the wells of 96-well plates. Then, the cells were incubated for 24 h. After incubation, the specimens of the scaffolds 5 × 5 × 3 mm were placed in the center of the wells. Then, the plates were incubated for 1, 3, 5, and 7 days. For biocompatibility testing, the first scaffolds, which were 5 × 5 × 3 mm, were placed in the center of the wells. Then, the cells were detached using trypsin/EDTA (0.25% *w*/*v* trypsin/0.02% EDTA, PanEco, Russia) and a 100 μL cell suspension, including 2000 cells, was placed on top of the experimental substrates that were positioned at the bottom of 96-well plate wells. The plates were incubated for 1, 3, 5, and 7 days. After incubation, the polymers samples were removed and placed into the wells with 100 μL α-MEM. In both cases, the cell viability was measured using the cell proliferation reagent XTT according to the manual (XTT Cell Proliferation Kit, Biological Industries, Israel) and the absorbance measurements were conducted at 450 nm with a reference wavelength at 620 nm using Zenyth 3100 Microplate Multimode Detector (Anthos Labtec Instruments GmbH, Salzburg, Austria).

### 3.6. In Vivo Experiments on Rats

The study was conducted on 21 adult Wistar rats. Keeping the laboratory animals and all manipulations with them, the study was carried out according to the ISO 10993-1:2009 ethical guidelines and approved by the Ethical Committee (protocol #10 dated 06.26.2020) of the Privolzhsky Research Medical University (Nizhniy Novgorod, Russia). The thirty-two-month-old male rats were sedated under general anesthesia using Zoletil-100 (0.05 mg/100 g) via intraperitoneal injection and laid in the lateral position. The wool was plucked around the surgical intervention at the left side of the back between the lobbies and hinder legs, the skin was treated with antiseptic, and the 8-mm-long incision was parallel to the backbone and 10 mm below it. Then, the skin was bluntly dissected forward at 10 mm. The scaffold was subcutaneously placed and the wounds were sutured. After 7, 14, 28, and 60 days, the animals were sacrificed, with a fragment of the soft tissue with the tested area being extracted and preserved in 10% formalin solution for histological examination [16].

### 3.7. Histological Study

The histological studies were carried out on days 7, 14, 28, and 60 after implantation. Following euthanasia, the polymer scaffolds (4 specimens for each tested material at each experimental time) were removed together with a surrounding soft tissue capsule. The explants were immersed in 4% formaldehyde in phosphate buffered saline (pH 7.4) and fixed for 24 h. Then, the specimens were prepared for histological examination as previously described [16]. The slices were stained using hematoxylin-eosin and analyzed under a light microscope Leica DM 2500 (Leica Microsystems, Germany). Up to 10 images (3132 × 2325 pixels) were taken from each specimen at ×100 and ×200 magnification and digitalized. Ten fields were counted in the response tissue of each sample. The fields were all tangential to the material.

### 3.8. Statistical Analysis

The statistical evaluation of the data was performed using the software package SPSS/PC+ Statistics™ 12.1 (SPSS). A non-parametric Kruskal–Wallis test was employed for all the statistical analyses. The data were averaged with the standard error to the mean (±SD) and considered significant for *p* < 0.05.

## 4. Conclusions

The technology of the pore formation using ammonium carbonate and sucrose as the porogens caused it to be possible to obtain the scaffolds of various shapes and microstructures. Flat porous structures, one side of which is porous and another smoother, are suitable for the in vitro cultivation of MSCs and 3T3 fibroblasts and can cause moderate inflammation with the formation of a fibrous capsule upon subcutaneous implantation. Other scaffolds are uniformly porous, and their pores are more structured. They are suitable for the in vitro culturing of fibroblasts, but not MSCs. Such scaffolds can be used to design more sophisticated devices, such as conduits from a dense polymer tube filled with a porous biomaterial (3D scaffold) for guided loose connective tissue sprouting. The obtained results can be applied to develop implantable medical devices for reconstructive surgery and regenerative medicine, such as dermal fillers, barrier membranes, conduits for guided tissue sprouting, and cell carriers.

## Figures and Tables

**Table 1:** Morphological features of porous scaffolds, *n* = 6.

Sample	Rupture Pores, μm	Lacunae, μm	Porosity, %
Side	External/Internal	External/Internal	
Scaffold-1	102.8 ± 37.3/7.2 ± 3.9	215.4 ± 137.0/43.1 ± 14.9	87.1 ± 7.1
Scaffold-2	41.1 ± 15.7	262.65 ± 125.54	94.2 ± 2.9

## Data Availability

Not applicable.

## Supplementary Materials

The following supporting information can be downloaded at: https://www.mdpi.com/article/10.3390/ijms24086965/s1, Reference [44] is cited in the Supplementary Materials.
