# Supplementary material for: Poly(3-hydroxybutyrate) 3D-Scaffold–Conduit for Guided Tissue Sprouting
Source: Int J Mol Sci. 2023 Apr 9;24(8):6965. doi: 10.3390/ijms24086965 (PMC10138660; doi:10.3390/ijms24086965)
Supplement: Supplementary file 1 [file ijms-24-06965-s001.zip › ijms-2068119-supplementary.pdf]

SUPPLEMENTARY MATERIALS

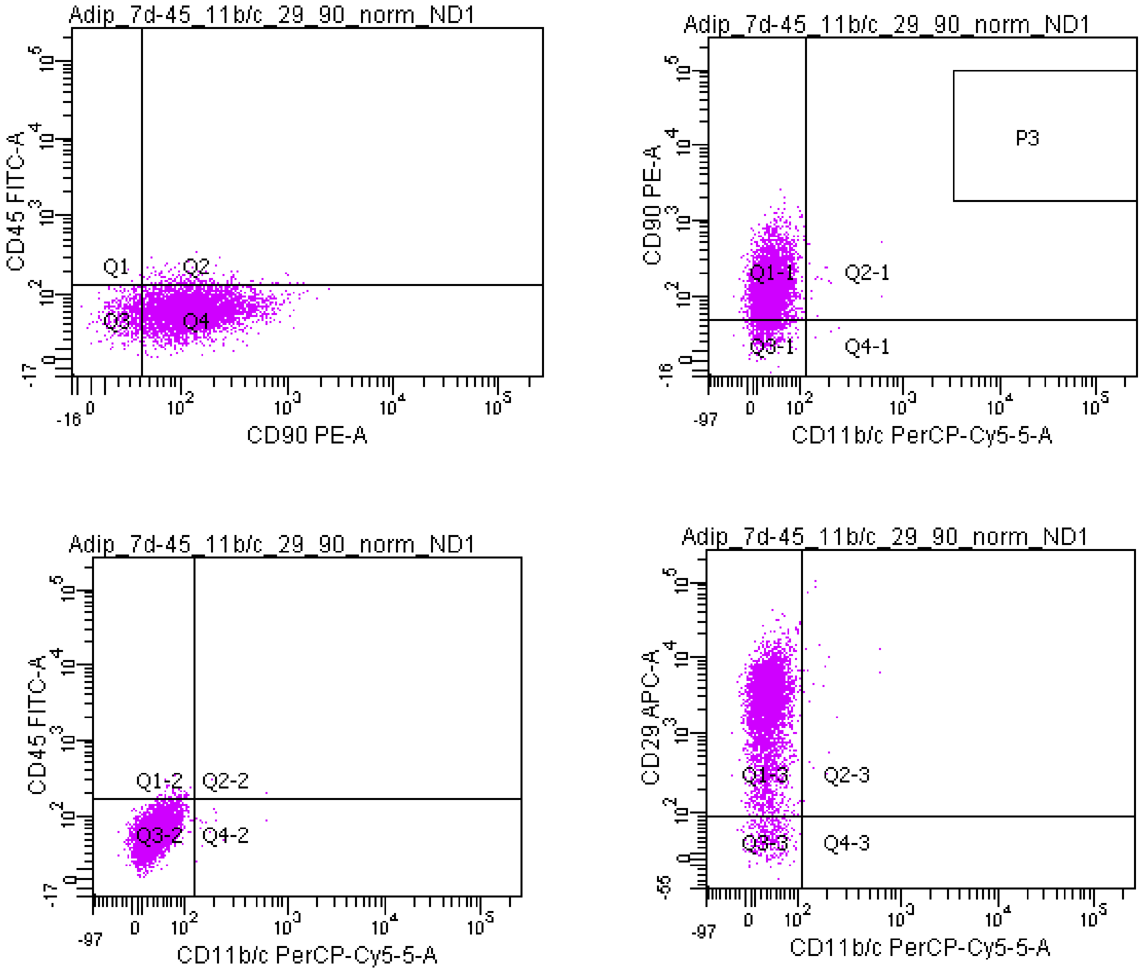

Figure S1. Data of flow cytometry of MSCs isolated from rat bone marrow.

Table S1. Morphological features of porous scaffolds, n = 6.

Sample	Melting temperature, T_m^{peak}, °C	Crystallinity degree, X_c, %
Scaffold-1	178	69
Scaffold-2	175	65

The crystallinity (X_c) of scaffolds was calculated by the following equation [44]:

$$X_c = (\Delta H_m + \Delta H_r) / \Delta H^0_m(\mathrm{PHB}) \times 100\%,$$

where ΔH_r and ΔH_m are the enthalpy changes caused by recrystallization and melting of the sample sample, respectively, ΔH^0_m(PHB) is the theoretical value for the thermodynamic enthalpy of melting that would be obtained for 100% crystalline PHB samples (146.6 J/g).

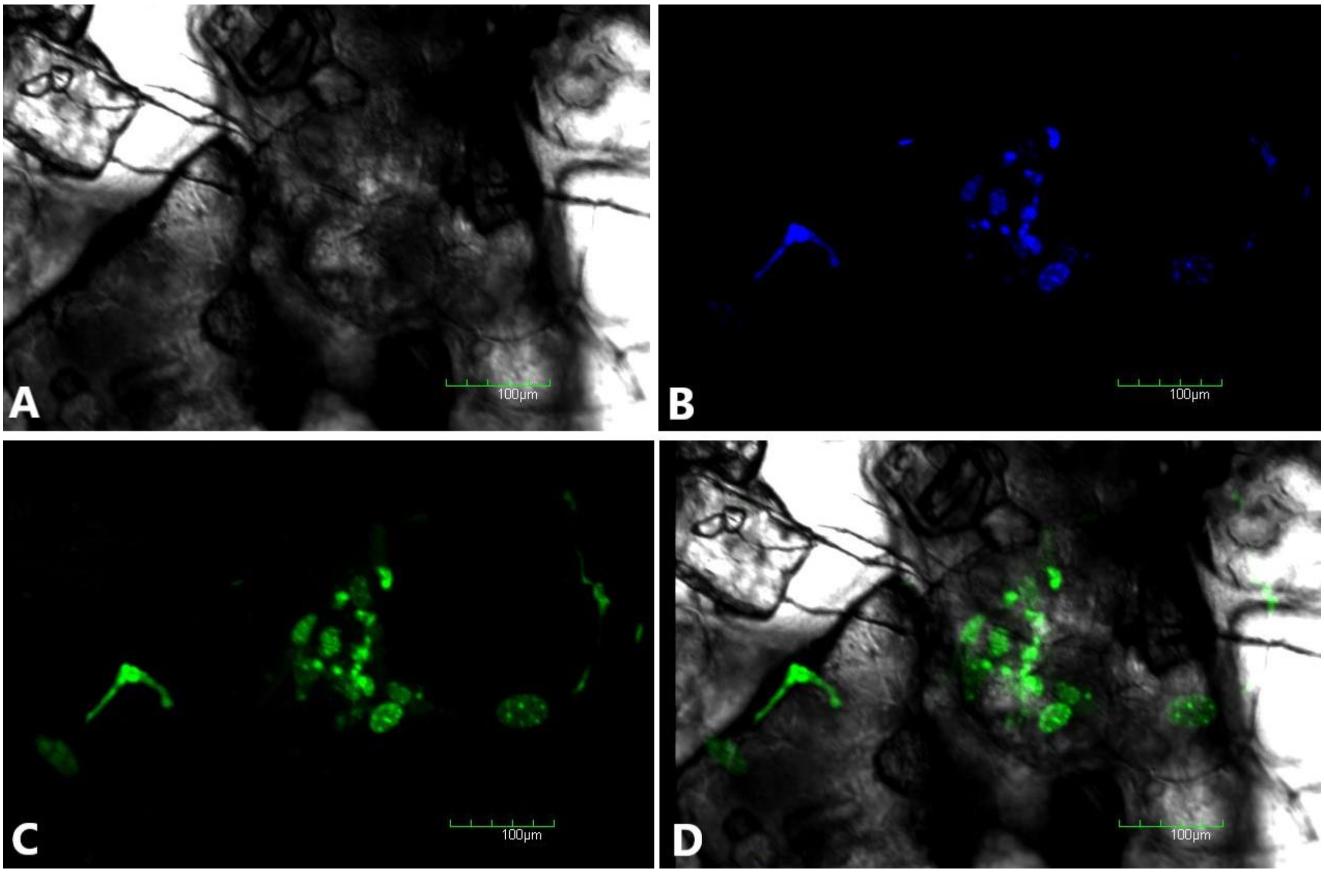

Figure S2. The Confocal laser scanning microscopy (CLSM) images of GFP-transfected MSCs attachment and growth on scaffolds-2: (A) light channel; (B) Hoechst dye blue fluorescence; (C) green fluorescence of GFP in MSCs; (D) Merged image of A and C.

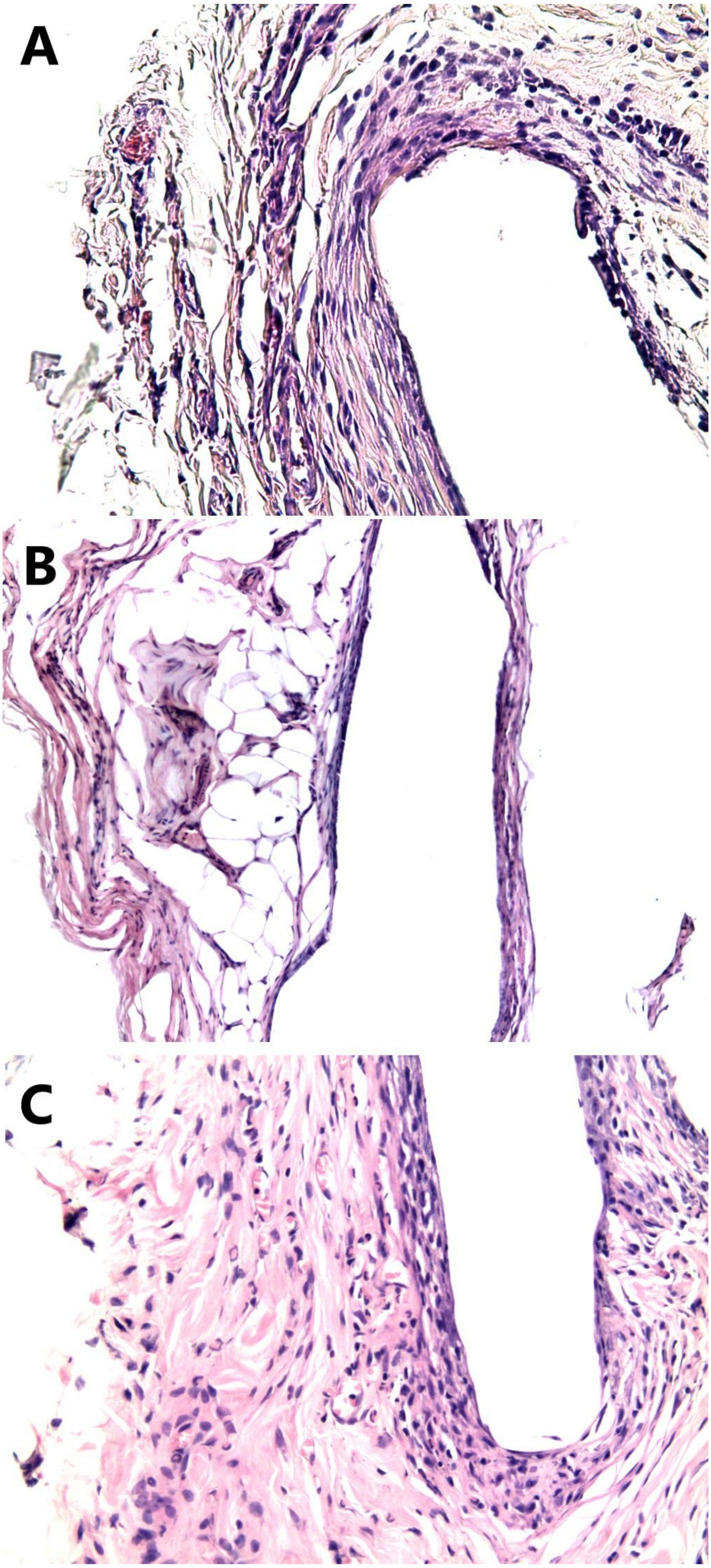

Figure S3. Histological section of PHB film on 7 (A), 14 (B) and 60 (C) day of subcutaneous implantation. The arrow shows the capsule formation. Hematoxylin - eosin staining; ×200.
